# Indications for Biological Treatment Combined with Allergen-Specific Immunotherapy: Who Is It Really Intended for?

**DOI:** 10.3390/jcm15187138

**Published:** 2026-09-14

**Authors:** Mirjana Turkalj, Ivana Banić

**Affiliations:** 1 Department of Allergy and Clinical Immunology, Srebrnjak Children’s Hospital, HR-10000 Zagreb, Croatia; 2 Faculty of Medicine, J.J. Strossmayer University of Osijek, HR-31000 Osijek, Croatia; 3 Faculty of Medicine, Catholic University of Croatia, HR-10000 Zagreb, Croatia; 4 Department of Medical Research, Srebrnjak Children’s Hospital, HR-10000 Zagreb, Croatia

**Keywords:** allergic diseases, allergen-specific immunotherapy, oral immunotherapy, biological treatment, monoclonal antibodies

## Abstract

Allergen-specific immunotherapy (AIT) is a well-proven treatment for allergic diseases and conditions such as Hymenoptera venom allergy, allergic rhinitis, allergic asthma, and, in recent years, food allergy. In order to enhance the efficacy and safety of allergen immunotherapy, biological agents such as omalizumab and dupilumab have been introduced. The use of omalizumab, in combination with AIT, has proven to be an effective option in treating patients with allergic asthma and during food desensitization, particularly in the dose escalation phase when adverse reactions are more common. To date, only a small number of well-structured clinical trials have been published on the combined use of biologics and AIT in patients with Hymenoptera venom allergy. Ongoing clinical studies are evaluating the efficacy of dupilumab as monotherapy or as an adjunct therapy to oral immunotherapy in patients with peanut allergy. Research is also underway on the use of dupilumab in patients with multiple food allergies and as an adjunct therapy to oral immunotherapy in patients with cow’s milk protein allergy. Further studies are needed to determine the optimal dosing and duration of biological therapy in combination with AIT, as well as to identify patients who may benefit most from such treatment regimes.

## 1. Introduction

Over the past century since it was introduced into clinical use, allergen immunotherapy (AIT) has undergone significant advancement, from standardization of allergen extracts and optimization of administration methods and routes to improvements in efficacy and safety. It remains the only etiological treatment for IgE-mediated allergic diseases and is likely one of the first implementations of personalized and precision medicine in clinical practice [1]. AIT has been proven effective in reducing allergic symptoms, decreasing the need for pharmacotherapy (particularly corticosteroids), and preventing disease exacerbation and progression [2,3].

Despite its proven efficacy, AIT still faces certain limitations, safety concerns, and contraindications in clinical practice [4,5,6]. Adverse reactions range from mild and localized to systemic and life-threatening. The risk is greater during the dose escalation phase and among patients with poorly controlled asthma or atopic dermatitis [4]. The safety and efficacy of AIT can be further improved by combining it with other medications or therapeutic interventions that modulate the type 2 immune response. One such strategy involves modulating stimulatory and inhibitory signals and cytokines involved in type 2 allergic inflammation with the use of biological agents [7,8].

**Figure 1:**
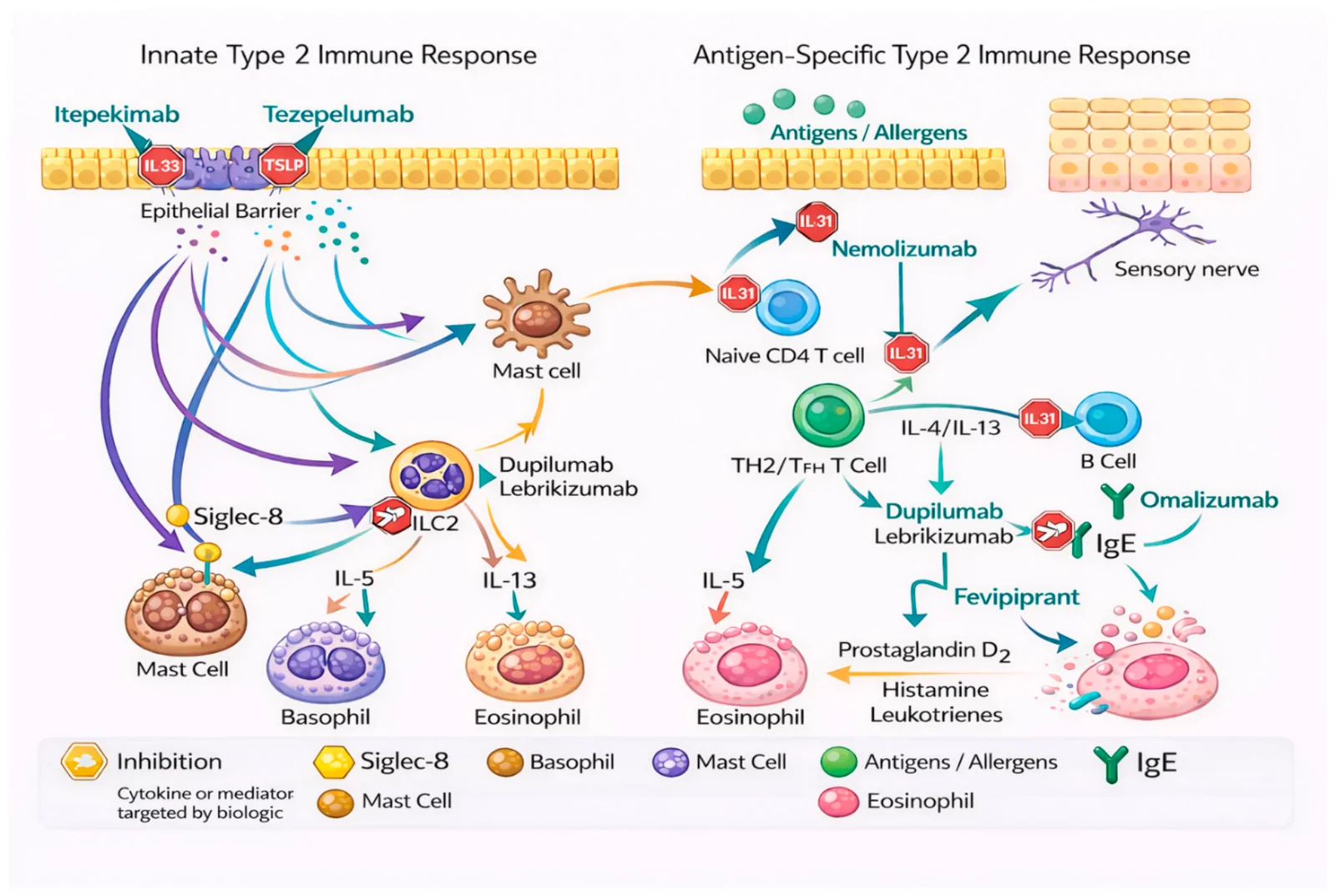
Schematic representation of common biological treatments aimed at mechanisms involved in the innate type 2 and antigen-specific (allergen-specific) type 2 immune responses, including the cytokine network and IgE synthesis. CD4—cluster of differentiation 4; IgE—immunoglobulin E; ILC2—type 2 innate lymphoid cells; IL-4—interleukin 4; IL-5—interleukin 5; IL-13—interleukin 13; IL-31—interleukin 31; IL-33—interleukin 33; Th2—type 2 helper T cells; T_FH_—follicular helper T cells.

Biological agents represent a type of treatment that specifically blocks or enhances biological phenomena, genes, or proteins involved in the pathophysiological mechanism of a given disease. The most commonly used biological treatments for allergic diseases such as asthma and atopic dermatitis are monoclonal antibodies directed against key pathophysiological targets, including IgE antibodies and proinflammatory cytokines [9]. The cytokine network, as well as common biologic and molecularly targeted treatments involved in the innate type 2 immune response mediated by interleukin 33 (IL-33), interleukin 25 (IL-25), and thymic stromal lymphopoietin (TSLP), and those involved in the antigen-specific type 2 immune response mediated by interleukin 4 (IL-4), interleukin 5 (IL-5), interleukin 13 (IL-13), and immunoglobulin E (IgE) synthesis, are illustrated in Figure 1.

With advances in understanding the immunological mechanisms of the adaptive type 2 immune response mediated by cytokines (IL-4, IL-13, and IL-5) and the innate response involving IL-25, TSLP, and IL-33, as well as IgE synthesis and immune memory maintenance, a new field of research has emerged focusing on targeted modulation of allergic inflammation with the use of monoclonal antibodies. By acting at different stages of the allergic inflammatory cascade, these therapeutics offer the potential to prevent the initiation and/or maintenance of the type 2 immune response, resulting in effective treatment of allergic diseases [9,10].

In the past 20 years, regimens that combine the use of AIT and biological agents were introduced to clinical practice to enhance the safety and long-term efficacy of treatment in allergic diseases [11]. Biological immunomodulators, when used in combination with AIT, may help avoid immediate or delayed adverse reactions, optimize treatment regimens, enhance therapeutic effects, and promote long-lasting tolerance to specific allergens, thereby improving the overall efficacy of immunotherapy. Moreover, the current contraindications for AIT, such as severe uncontrolled asthma, may be overcome by using biological therapy to control certain type 2 inflammatory mediators in patients with severe atopy [12,13].

## 2. Mechanisms of AIT and Biological Treatment

In allergic inflammation, effector cells such as mast cells, T and B cells, eosinophils, and dendritic cells play key roles and communicate through a complex cytokine network. During AIT, mechanisms by which these cells act and produce cytokines are modulated. AIT induces allergen-specific tolerance by acting on regulatory T lymphocytes (Tregs), which release transforming growth factor β (TGF-β) and interleukin-10 (IL-10). These cytokines promote immune skewing toward a Th1-dominant response, characterized by the secretion of interferon γ (IFN-γ). The inhibitory effects of IL-10, TGF-β, and IFN-γ on Th2 cytokines (IL-4 and IL-5) suppress the activity of T-helper type 2 (Th2) cells, as well as type 2 innate lymphoid cells (ILC2s), mast cells, basophils, eosinophils, and their mediators [14].

Additionally, IL-10, TGF-β, and IFN-γ stimulate the production of allergen-specific immunoglobulin G subclass 4 (sIgG4) antibodies, which compete with allergen-specific IgE for receptor binding, thereby preventing IgE-dependent histamine release from mast cells. Consequently, mast cell activation and degranulation are reduced, preventing anaphylaxis, while the interaction of IgE with low-affinity receptors (FcεRIIb) on B cells involved in allergen presentation to T cells is impeded. Moreover, AIT inhibits ILC2s, which contribute to allergic inflammation through the production of type 2 cytokines following activation by epithelial-derived cytokines such as TSLP, IL-25, and IL-33 [14,15].

Specific monoclonal antibodies (biologic agents) can interact with cellular and cytokine networks by effectively disrupting signaling pathways activated in these cells, thereby mitigating the inflammatory immune response. Dupilumab is a human monoclonal antibody that binds to the IL-4 receptor alpha (IL-4Rα), inhibiting both IL-4 and IL-13 signaling [16,17]. Omalizumab binds to free IgE, preventing its interaction with high-affinity IgE receptors (FcεRI) on mast cells and basophils [18]. Benralizumab, reslizumab, and mepolizumab act on IL-5 or its receptor, thereby interfering with eosinophil activation and survival [19]. Tezepelumab is a TSLP-neutralizing monoclonal antibody approved for the treatment of asthma and is currently being investigated as an adjunct to allergen immunotherapy using cat epithelium extracts for tolerance induction [20]. Etokimab, a humanized monoclonal antibody specific for IL-33, is also being studied in combination with allergen immunotherapy in patients with food allergies [21].

## 3. Use of Biologicals and AIT in Patients with Asthma and Allergic Rhinoconjunctivitis

Some of the earliest studies involving biologicals evaluated the efficacy of combined omalizumab and AIT in respiratory allergies [22,23]. Omalizumab has proven effective in treating severe allergic asthma and allergic rhinitis (AR). The primary goals of using omalizumab during AIT include the following:

- Enabling AIT-mediated tolerance in patients who are unable to achieve allergen dose escalation during the early build-up phase due to pronounced immunoreactivity.
- Minimizing the risk of allergic adverse events associated with AIT, particularly in patients with bronchial asthma, which is a known risk factor for adverse events.

Studies in patients with asthma reported that combined treatment with AIT and omalizumab resulted in a significant decrease in asthma symptoms and an increase in the patients’ quality of life compared with AIT alone [24,25]. Omalizumab has also been used to improve the safety of AIT and reduce the risk of adverse events [19,20,25], especially in accelerated or “rush” AIT protocols, which are likely more hazardous than standard regimens. A study involving patients with allergic rhinitis on a rush protocol for ragweed omalizumab showed pretreatment with omalizumab before AIT resulted in fewer adverse events and a 5-fold decrease in the risk of rush protocol-associated anaphylaxis compared to patients receiving AIT alone [26]. A study involving high-risk patients with persistent asthma with an inadequate response to inhaled corticosteroid treatment receiving subcutaneous immunotherapy (SCIT) demonstrated that severe adverse events were significantly lower in patients treated with omalizumab compared to those receiving placebo. Additionally, a greater proportion of patients treated with omalizumab was able to reach the target immunotherapy maintenance dose compared to the placebo group [27].

In addition to improving the safety and efficacy of AIT when combined with a biological therapy approach, studies have also demonstrated a beneficial effect on reducing the cumulative steroid dose as a secondary outcome in asthma management. Lambert et al. [28] and Stelmach et al. [29] showed a reduction in maintenance steroid doses in children with severe asthma who underwent AIT after achieving adequate disease control with omalizumab.

Although severe asthma is currently considered a contraindication for AIT, recent studies indicate that it can be successfully utilized when combined with biologic therapies. Valdesoiro-Navarrete et al. demonstrated that using omalizumab as a pretreatment to achieve asthma control before initiating AIT led to significantly better asthma control, lung function, and quality of life in children compared to AIT monotherapy [30]. Similarly, Massanari et al. showed that omalizumab effectively mitigates systemic and severe allergic reactions to AIT for perennial aeroallergens (cat, dog, and house dust mite) in patients with an inadequate response to inhaled corticosteroids (ICS) [27]. Furthermore, this combination enabled a higher percentage of patients to successfully reach their target immunotherapy maintenance dose. Additionally, Bożek et al. confirmed that combining AIT (using standardized house dust mite extracts) with omalizumab was more effective at reducing symptoms and decreasing daily ICS reliance than either omalizumab or AIT alone [31]. Consequently, for patients whose poor asthma control would otherwise contraindicate AIT, omalizumab pretreatment can facilitate safe administration.

Studies combining biological treatment with AIT in patients with allergic rhinitis (AR) were first conducted in the early 2000s. A study involving children and adolescents with AR showed that patients on both omalizumab and AIT had a 48% reduction in allergen-induced symptom load during two pollination seasons (birch and grass) [32]. Additionally, another pediatric study demonstrated that combined treatment with omalizumab and AIT reduced the number of symptomatic days, use of rescue medications, and overall symptom severity, which were all significantly lower in this group compared with those receiving AIT or biological treatment alone [33].

Dupilumab is currently approved for the treatment of atopic dermatitis (eczema), asthma, and chronic rhinosinusitis with nasal polyps [34]. In a 16-week trial, dupilumab was evaluated as monotherapy or as an adjunct to subcutaneous immunotherapy (SCIT) for patients allergic to grass pollen. Although the combined treatment with dupilumab and SCIT did not significantly reduce the total nasal symptom score, compared with SCIT alone, it improved SCIT tolerability, reduced adverse events, lowered the need for epinephrine as rescue medication, and increased the proportion of patients who reached the target maintenance dose compared to SCIT monotherapy [35]. In another study involving patients with seasonal AR on combined treatment with dupilumab and AIT to Timothy grass, this combination also did not reduce symptom scores but did improve tolerability of SCIT up-titration. Moreover, this study showed that dupilumab combined with SCIT significantly increased the levels of allergen-specific IgG4, as well as the sIgG4/sIgE ratio compared to SCIT alone [36].

However, the role of biologics as an adjunct to AIT in patients with allergic rhinitis remains questionable. Although available studies suggest that biologic therapy may improve the tolerability and safety of AIT, particularly during the build-up phase, evidence for an additional clinical benefit in terms of symptom reduction remains limited and inconsistent. Therefore, the routine use of biologics in combination with AIT for allergic rhinitis cannot currently be considered a well-established indication and should be reserved for selected patients based on individual clinical circumstances.

## 4. Use of Biologics and AIT in Patients with Food Allergy

Food allergy is an increasingly significant public health concern. It may present with mild symptoms or progress to life-threatening or even fatal anaphylaxis and greatly impair the patients’ and their families’ quality of life. Due to lack of standardization and risk for severe anaphylactic reactions, the use of AIT in patients with food allergies has not yet been introduced into routine clinical practice in most healthcare centers. Oral immunotherapy with whole foods (OIT) is currently the most commonly used method, while the standardized peanut OIT formulation remains the only one approved for use in children and adolescents [37]. However, adverse reactions occur much more frequently during OIT than in patients undergoing AIT for respiratory allergies, in patients allergic to Hymenoptera venom, and in other AIT routes and regimens for food allergy [38]. In a meta-analysis evaluating the efficacy and safety of OIT in patients with peanut allergy, OIT proved effective in achieving desensitization and increasing the threshold of tolerance, but it significantly increased the risk of allergic and anaphylactic reactions [39]. For this reason, combination treatment with biologics was introduced before or during OIT in high-risk patients.

Omalizumab is most commonly used as an adjunct during OIT. In a study by Nadeau et al., children with a cow’s milk allergy underwent rapid oral milk desensitization in combination with omalizumab prior to and up to 16 weeks of OIT, until achieving a maximum dose of 2000 mg of cow’s milk protein [40]. A total of 82% of the patients tolerated the maximum milk protein dose, and moreover, desensitization was maintained in all patients with continued milk intake after omalizumab discontinuation.

Since then, multiple clinical trials have evaluated the use of omalizumab in combination with OIT for peanut, egg, and milk allergies, as well as multiple allergens simultaneously [41,42]. Studies have shown a significant increase in the tolerated dose for different foods after an oral food challenge to cow’s milk and eggs for patients on OIT alongside omalizumab for 13 weeks compared to pretreatment with omalizumab, pre-omalizumab, and in patients with severe food allergy receiving combined treatment with omalizumab and OIT for 24 weeks [43]. In recent years, the combination of biologics and OIT has been used for multifood protocols, demonstrating safe and effective rapid desensitization with high maintenance rates. In a study involving children with food allergies to two to five allergens, the combined treatment of omalizumab with multifood OIT for 16 weeks (4 weeks omalizumab pretreatment and co-treatment for 12 weeks) was associated with greater tolerance to up to 2 g of each culprit food, with fewer adverse events compared with OIT alone [44].

Beyond anti-IgE treatment, the efficacy and safety of dupilumab as an adjunct to OIT and as monotherapy for food allergy are being investigated. A phase 2 study involving children with peanut allergy showed that combined treatment with dupilumab and OIT resulted in a higher number of patients who passed a double-blind placebo-controlled (DBPC) food challenge test to a cumulative dose of just over 2 g of peanut protein compared to children on OIT alone, including after the maintenance period was over. However, co-treatment with dupilumab did not increase the safety of OIT [45]. A phase 2 randomized double-blind trial is also underway to assess the effects of dupilumab as an adjunct to OIT for cow’s milk protein allergy. In this trial, patients in the treatment group receive dupilumab before and during the OIT dose escalation phase (16 weeks in total), followed by eight weeks of OIT without dupilumab. The proportion of patients able to tolerate at least 2040 mg of milk protein will be assessed in week 18 of treatment [46].

Biologics targeting upstream alarmins, such as IL-25, IL-33, and TSLP, represent additional therapeutic options for food allergy. Elevated levels of IL-4, IL-5, and IL-13 lead downstream to a shift from a tolerant, Th1-dominant response toward a pro-allergic Th2 response [9]. These alarmins are critical for initiating and maintaining food allergy as they play a central role in sustaining the Th2 response [47]. Etokimab is a humanized immunoglobulin G subclass 1 (IgG1)/kappa monoclonal antibody developed to bind IL-33 and neutralize its biological activity [48]. In a phase 2a randomized DBPC clinical trial involving adult patients with peanut allergy, a single dose of etokimab significantly increased peanut tolerance (up to 275 mg of peanut protein) and was associated with fewer adverse events. Etokimab administration also resulted in reduced wheal size in skin prick tests and decreased peanut-specific IgE after 14 days. Moreover, in the etokimab group, clinical improvements correlated with reductions in IL-4, IL-5, interleukin 9 (IL-9), and IL-13 expression in T-helper cells [49]. A new ongoing phase 2 DBPC clinical trial in children and adults with peanut allergy aims to evaluate the safety and efficacy of the combined use of tezepelumab and OIT. The study plan includes 8 weeks of tezepelumab monotherapy followed by 56 weeks of combined treatment with tezepelumab and OIT and a DBPC food challenge test 12 weeks after treatment cessation (up to a 144 mg cumulative dose of peanut protein) [50].

While biological agents are currently used to treat diseases associated with eosinophilic inflammation, such as eosinophilic asthma, their efficacy in treating eosinophilic gastrointestinal disorders, particularly eosinophilic esophagitis (EoE), remains elusive. Dupilumab has already been approved as monotherapy for EoE, and other biologics, such as anti-IL5 and anti-IL13 pathway biological agents, are increasingly being studied for treatment of EoE [51,52]. Interestingly, both oral and sublingual immunotherapy have been accompanied by the occurrence of eosinophilic esophagitis as a treatment-related adverse event in certain patients [53]. The potential use of biologic agents as adjuncts to immunotherapy in the management of EoE is an area of growing research interest [54].

## 5. Use of Biologics and AIT in Patients with Insect Venom Allergy

Venom immunotherapy (VIT) remains the only disease-modifying intervention for patients with allergy to Hymenoptera venom, exhibiting high levels of efficacy in inducing immune tolerance and significantly improving the patient’s quality of life. However, adverse reactions are common, both in the dose escalation and maintenance phases, and may be recurrent. These include local injection site reactions (redness, swelling), as well as systemic allergic manifestations such as generalized urticaria, angioedema, bronchospasm, and anaphylaxis [55]. Antihistamines have proven effective in preventing mild hypersensitivity reactions; however, pretreatment with omalizumab has been shown to be beneficial in cases of repeated severe adverse reactions that prevent reaching the full maintenance dose [56].

Most studies investigating the use of omalizumab have demonstrated a good safety profile in protecting high-risk patients (e.g., those with mast cell disorders such as mastocytosis or mast cell activation syndrome, as well as highly sensitized beekeepers or individuals with recurrent anaphylaxis during VIT) from severe reactions during the desensitization process up to tolerance achievement, regardless of the immunotherapy regimen (conventional rush and ultra-rush protocols), source of allergen (honeybee, wasp), and omalizumab dosing (one-time, bi-weekly, or monthly) [56,57,58,59,60,61]. Conversely, several studies reported failure to achieve tolerance due to anaphylactic reactions despite the use of omalizumab [62,63]. The evidence supporting the combined use of biologics and AIT is heterogeneous, with the strength of evidence varying considerably across clinical indications. This limitation is particularly relevant to Hymenoptera venom immunotherapy, where most evidence for omalizumab consists of small case reports and case series, with substantial variations in patient characteristics, omalizumab dosing, treatment duration, and VIT protocols. Consequently, the observed benefits may not be readily generalizable, and the absence of adequately powered randomized controlled trials limits firm conclusions regarding efficacy, optimal treatment regimens, and long-term outcomes. The available retrospective evidence further suggests that although omalizumab may facilitate achievement of the maintenance dose in selected high-risk patients, it may not exert an additional immunomodulatory effect on VIT or provide a definitive long-term solution [64]. Therefore, these findings should be interpreted cautiously, and standardized prospective studies are needed to establish the role of biologic therapy as an adjunct to VIT.

Antihistamines may also be used as adjunctive premedication during AIT, particularly in patients who develop recurrent local or mild systemic cutaneous reactions. Evidence from randomized controlled trials indicates that antihistamine pretreatment can reduce the frequency and severity of local reactions and some systemic reactions and may facilitate dose escalation and achievement of the target maintenance dose [65]. A meta-analysis of 11 randomized controlled trials involving 609 patients demonstrated that antihistamine pretreatment significantly reduced both the frequency of systemic adverse reactions and moderate-to-severe systemic reactions while also increasing the likelihood of reaching the target maintenance dose [66]. In venom immunotherapy, pretreatment with H1-antihistamines has similarly been shown to reduce large local reactions and, to some extent, systemic adverse events, and is recommended by EAACI [65]. Antihistamines may therefore be particularly useful when adverse reactions interfere with dose escalation or adherence to AIT. However, antihistamine premedication does not eliminate the risk of systemic reactions or anaphylaxis and should not be considered a substitute for appropriate patient selection, dose adjustment, monitoring, or emergency treatment [65].

Compared with biologics, antihistamines have a more limited role in AIT. Their effects are primarily symptomatic, and they focus on reducing histamine-mediated local and cutaneous reactions, whereas biologics such as omalizumab act upstream in the allergic cascade and may provide a broader protective effect against systemic reactions. Thus, antihistamines are generally considered a relatively simple adjunct to improve tolerability, whereas biologic therapy may be considered in carefully selected patients in whom AIT cannot otherwise be safely initiated or maintained.

## 6. Conclusions

The combination of biologic therapy and AIT represents a highly effective treatment strategy for high-risk patients and individuals prone to severe adverse reactions. This dual approach is increasingly applied across diverse clinical indications, although there are specific settings where the rationale/evidence is stronger. For example, in asthma, food allergy, and insect venom allergy, the combined use of biologics and AIT synergistically enhances both the efficacy in achieving tolerance and the safety profile of AIT. Table 1 summarizes the most common indications for the combined use of biologics and AIT [27,44,56,65,67,68,69,70].

**Figure 2:**
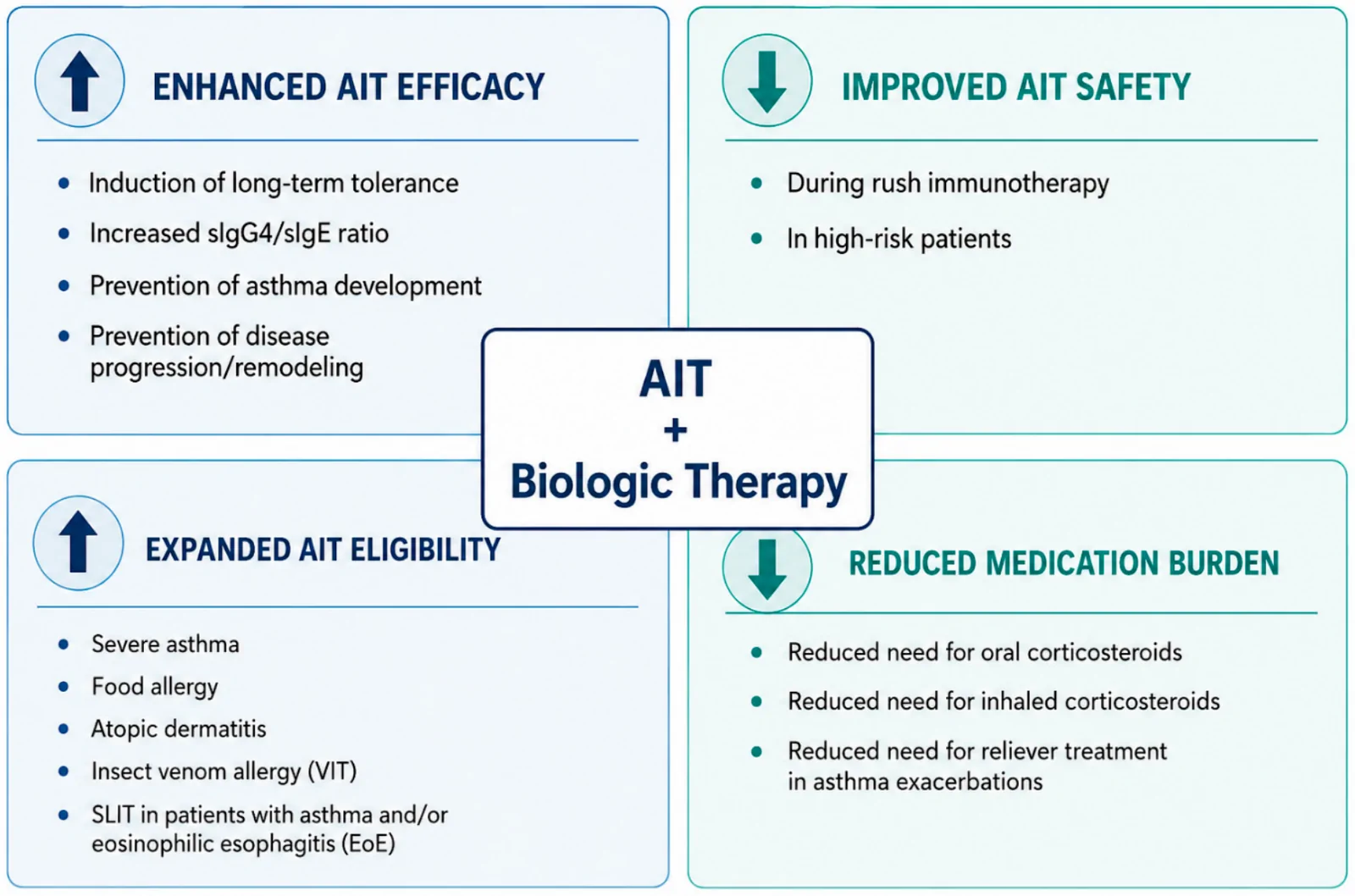
Graphic summary of the combined use of biologics and allergen-specific immunotherapy: advantages and indications. AIT—allergen-specific immunotherapy; EoE—eosinophilic esophagitis; sIgE—allergen-specific immunoglobulin E; sIgG4—allergen-specific immunoglobulin G subclass 4; SLIT—sublingual immunotherapy; VIT—venom immunotherapy.

Furthermore, evidence suggests that the combined use of biologic agents and AIT may induce profound, long-term immunomodulation, thereby sustaining the suppression of allergic symptoms even after treatment cessation (Figure 2).

Despite these clinical advantages, several critical gaps must be addressed before widespread implementation of the combined use of biologics and AIT can be achieved, as a definitive consensus is currently lacking regarding the following:

- The optimal dosing regimens and duration of biologic therapy when utilized as an adjunct to AIT;
- Robust patient selection criteria to identify individuals who will derive the greatest therapeutic benefit within each specific indication;
- The long-term safety profile and potential risks associated with prolonged, combined exposure;
- The overall cost-effectiveness and socioeconomic viability of this dual approach.

A recent systematic review of economic evaluations in allergic rhinitis found that the majority of published studies considered both subcutaneous and sublingual AIT to be cost-effective compared with symptomatic pharmacotherapy, although substantial heterogeneity existed between countries, healthcare systems, treatment modalities, and economic modelling assumptions [72,73]. In contrast, biologic therapies generally have substantially higher acquisition costs, and their cost-effectiveness varies according to the specific biological features, indication, disease severity, treatment response, healthcare system, and willingness-to-pay threshold [74]. However, there is currently limited direct evidence evaluating the cost-effectiveness of combining biologic therapy with AIT compared with AIT alone or alternative treatment strategies. The economic value of the combined approach therefore depends not only on the acquisition cost of the biologic, but also on whether its use reduces systemic adverse reactions, facilitates achievement of the maintenance dose, improves adherence, reduces healthcare utilization, or enables successful AIT in patients who would otherwise be unable to tolerate treatment. These potential benefits may offset part of the additional cost in carefully selected high-risk patients, but this remains insufficiently demonstrated in formal health-economic evaluations. Consequently, the cost-effectiveness of combined biologic and AIT treatment is likely to be highly context-dependent and may differ substantially between countries according to drug pricing, reimbursement policies, healthcare resource utilization, and willingness-to-pay thresholds. Prospective economic evaluations incorporating long-term clinical outcomes, quality-adjusted life-years, healthcare utilization, treatment discontinuation, and indirect costs are needed to determine which patient populations derive sufficient additional benefit to justify the costs of combined therapy. In summary, further research in this field is crucial to unlock the full therapeutic potential of this combined modality and to refine and improve the current management paradigms in allergic diseases. Finally, resolving these mechanistic and clinical uncertainties will pave the way for standardized, biomarker-driven AIT protocols that will help transition this innovative approach into routine precision medicine.

## Figures and Tables

**Table 1:** Proposed indications and clinical characteristics of patients who may be considered for combined biological therapy and allergen-specific immunotherapy and the current level of evidence for such treatment use.

Clinical Indication	Proposed Candidate Profile	Potential Rationale for Combined Treatment	Current Level of Evidence
Severe allergic asthma	Patients with severe, uncontrolled allergic asthma despite optimized guideline-directed therapy, in whom AIT is otherwise indicated; particularly those considered at increased risk of systemic reactions during AIT or in whom asthma control limits the safe administration of AIT.	Biologic therapy, particularly anti-IgE treatment, may improve asthma control and reduce the risk of systemic reactions during AIT, potentially allowing patients to reach and maintain the target AIT dose.	Moderate evidence for improved AIT tolerability; limited evidence for defining long-term benefits of the combination. A randomized trial of omalizumab pretreatment demonstrated fewer systemic reactions and a greater proportion of patients reaching the target maintenance dose [27].
Hymenoptera venom allergy (VIT)	Patients with a clear indication for VIT who experience recurrent systemic reactions during VIT despite appropriate protocol modification, premedication, and dose adjustment, particularly those in whom the maintenance dose cannot be achieved; consideration may be especially relevant in patients with severe previous sting reactions or other risk factors for severe reactions.	Omalizumab may suppress IgE-mediated reactions during VIT and facilitate achievement of the maintenance dose in patients who otherwise cannot tolerate VIT.	Low-to-moderate evidence; predominantly case reports, case series, and retrospective studies. EAACI guidelines establish VIT as the treatment of choice for preventing systemic sting reactions but do not establish biologic therapy as routine adjunctive treatment [56]. Small studies suggest that omalizumab can facilitate VIT in patients with recurrent systemic reactions [67,68].
IgE-mediated food allergy/food OIT	Patients with confirmed IgE-mediated food allergy who are appropriate candidates for OIT but have a high risk of adverse reactions, low reaction thresholds, multiple food allergies, or difficulty initiating/up-dosing OIT; treatment should be undertaken in experienced specialist centers.	Omalizumab may reduce adverse reactions during OIT and facilitate more rapid or successful dose escalation, potentially expanding the feasibility of OIT in selected high-risk patients.	Moderate and rapidly evolving evidence. Randomized trials have demonstrated improved safety and/or facilitated desensitization with omalizumab-assisted OIT [44,69]. The 2024 EAACI food allergy guideline conditionally suggests omalizumab for IgE-mediated food allergy, although the evidence and licensing differ between countries [70,71].
Allergic rhinitis	Patients with persistent, clinically significant allergic rhinitis who have a clear indication for AIT but experience substantial adverse reactions or poor tolerability during SCIT; particularly selected patients with relevant comorbid type 2 disease for which biologic treatment is independently indicated.	Biologics may improve tolerability of AIT during the build-up phase; however, evidence for additional improvement in rhinitis symptoms beyond AIT remains limited.	Low/insufficient evidence for routine combined treatment. Studies of omalizumab and dupilumab suggest improved SCIT tolerability in selected patients, but consistent additional improvement in rhinitis outcomes has not been demonstrated. AIT remains the established disease-modifying treatment for appropriately selected allergic rhinitis patients [65].

## Data Availability

No new data were created or analyzed in this study. Data sharing is not applicable to this article.

## Abbreviations

The following abbreviations are used in this manuscript:

AIT	allergen-specific immunotherapy
AR	allergic rhinoconjuctivitis/rhinitis
DBPC	double-blind placebo-controlled
EoE	eosinophilic esophagitis
FcεRIIb	low-affinity immunoglobulin E receptor
FcεRI	high-affinity immunoglobulin E receptor
IFN-γ	interferon gamma
IgE	immunoglobulin E
IgG1	immunoglobulin G subclass 1
IL-4	interleukin 4
IL-4Rα	interleukin 4 receptor subunit alpha
Il-5	interleukin 5
IL-9	interleukin 9
IL-10	interleukin 10
IL-13	interleukin 13
IL-25	interleukin 25
IL-33	interleukin 33
ILC2	type 2 innate lymphoid cells
OIT	oral immunotherapy
SCIT	subcutaneous immunotherapy
TGF-β	transforming growth factor β
Th1	type 1 T helper cells
Th2	type 2 T helper cells
Treg	regulatory T cells
TSLP	thymic stromal lymphopoietin
VIT	venom immunotherapy
